# The Red Back’s Birthday

**Published:** 1905-07

**Authors:** 


### TEXAS MEDICAL JOURNAL.

AUSTIN, TEXAS.

A MONTHLY JOURNAL OF MEDICINE AND SURGERY.

EDITED AND PUBLISHED BY
F. E. DANIEL, M. D.
Mrs. F. E. DANIEL, Business Manager.

Published Monthly at Austin, Texas. Subscription price $1.00 a year in advance

THE RED BACK'S BIRTHDAY.—Twenty years ago today the *Texas Medical Journal* was launched. It has been a success, gratifying at once to its founder and its hosts of loyal friends and supporters. We start on our twenty-first round stimulated and encouraged to continue with renewed vigor the "campaign of education," and the war upon quacks, quackery and all sorts of jerrymandering and crookedness; with the courage to attack it in high places, as well as in low. In returning thanks for and asking the continuation of the liberal support my efforts have ever had—and for fear of misapprehension because of intentional misrepresentation by certain enemies, some of whom have deadbeat me out of subscription dues —I want to say: The *Journal* earnestly advocates organization, the building up, cohesion and harmony of our State Association, but not by means of intimidation, threats or coercion of those who, for reason of their own, do not want to join. It should be voluntary, or not at all, and all physicians of standing are invited to come in.

*This was read at the Portland meeting of medical editors.

